# Amorphous boron nitride: synthesis, properties and device application

**DOI:** 10.1186/s40580-025-00486-1

**Published:** 2025-05-02

**Authors:** Seyed Mehdi Sattari-Esfahlan, Saeed Mirzaei, Mukkath Joseph Josline, Ji-Yun Moon, Sang-Hwa Hyun, Houk Jang, Jae-Hyun Lee

**Affiliations:** 1 Institute for Microelectronics, 1040 Vienna, TU Austria; 2 CEITEC BUT, Brno University of Technology, Purkynova 123, 61200 Brno, Czech Republic; 3 Fraunhofer Institute for Material and Beam Technology, WinterbergstraBe 28, E01277 Dresden, Germany; 4 Department of Energy Systems Research, Ajou University, Suwon, 16499 Republic of Korea; 5 Department of Mechanical Engineering and Materials Science, Washington University in St. Louis, St. Louis, MO 63130 USA; 6 Department of Electrical and Computer Engineering, Sungkyunkwan University, Suwon, 16419 Republic of Korea; 7 Center for Functional Nanomaterials, Brookhaven National Laboratory, Upton, New York, 11973 USA; 8 Institute for Functional Intelligent Materials, National University of Singapore, Singapore, 117575 Singapore

## Abstract

Amorphous boron nitride (a-BN) exhibits remarkable electrical, optical, and chemical properties, alongside robust mechanical stability, making it a compelling material for advanced applications in nanoelectronics and photonics. This review comprehensively examines the unique characteristics of a-BN, emphasizing its electrical and optical attributes, state-of-the-art synthesis techniques, and device applications. Key advancements in low-temperature growth methods for a-BN are highlighted, offering insights into their potential for integration into scalable, CMOS-compatible platforms. Additionally, the review discusses the emerging role of a-BN as a dielectric material in electronic and photonic devices, serving as substrates, encapsulation layers, and gate insulators. Finally, perspectives on future challenges, including defect control, interface engineering, and scalability, are presented, providing a roadmap for realizing the full potential of a-BN in next-generation device technologies.

**Graphical Abstract:**

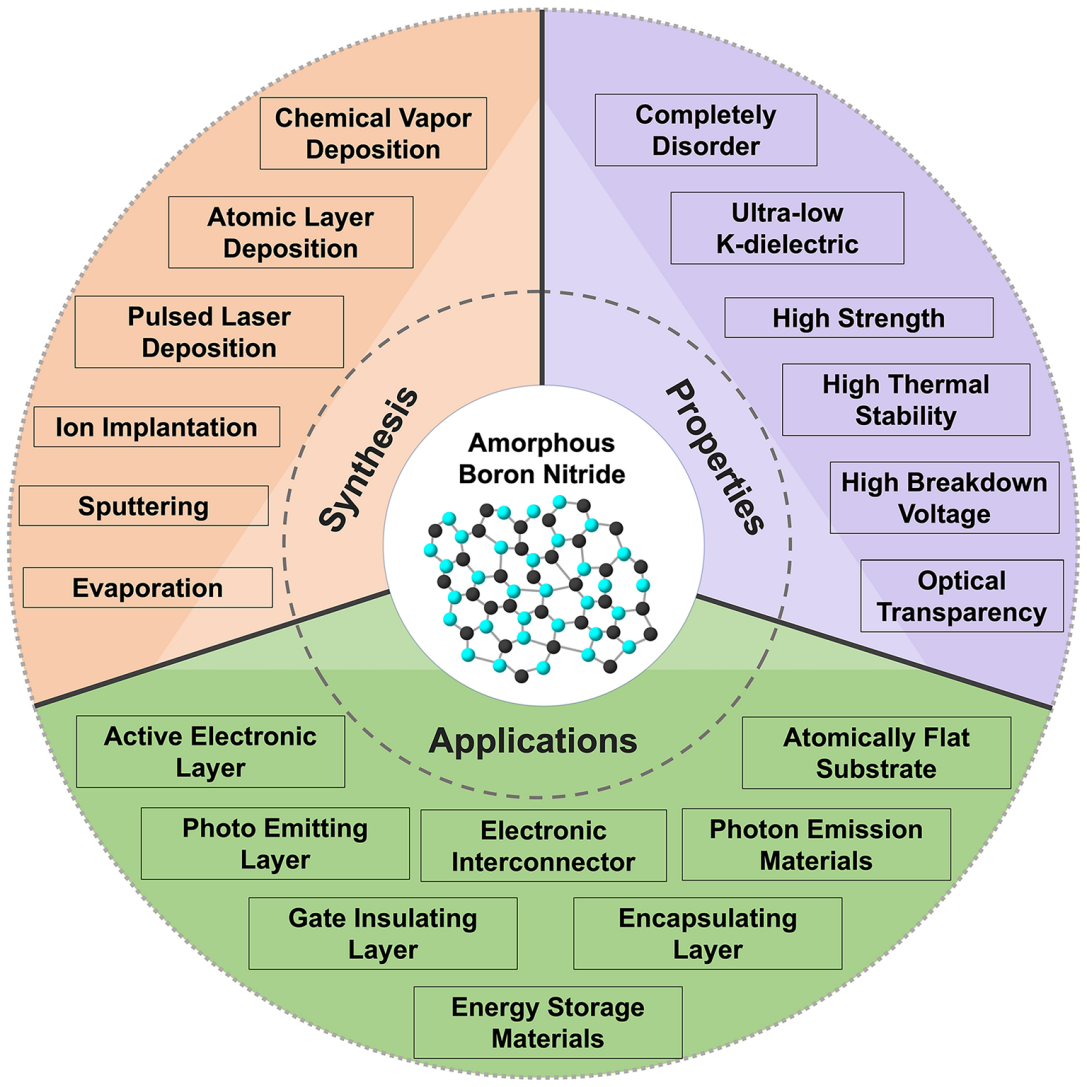

## Introduction

Boron nitride (BN) is one of the most successful insulating 2D materials offering a range of versatile features that make it highly attractive for various applications [1–4]. Its exceptional material properties such as robust mechanical strength, high thermal and chemical stability, large bandgap, smooth surface morphology, and low dielectric constant demonstrate significant potential across numerous technological domains [1, 4–15]. BN materials are categorized into an amorphous phase and three crystalline phases: hexagonal (h-BN), cubic (c-BN), and wurtzite (w-BN) [16, 17]. The properties of BN materials closely resemble the isoelectronic forms of their carbon counterparts. h-BN possesses a honeycomb structure akin to graphene, while c-BN features a cubic structure similar to diamond [18].

Among these crystalline phases, c-BN is particularly noted for its extreme hardness, high thermal conductivity, elevated melting point, and remarkable chemical inertness due to its compact atomic structure. Conversely, w-BN represents a metastable phase of BN, and its structural stability remains a topic of discussion. Crystalline h-BN consists of atomically flat layers of alternating hexagonal B and N atoms, held together by van der Waals (vdW) bonds, features sp^2^ hybridized and highly polarized B-N covalent bonds along the plane. Unlike graphene, h-BN’s ionic bonding nature introduces significant polarity, making it highly anisotropic and suitable for diverse applications across multiple disciplines [19, 20].

Ultra-thin h-BN has garnered significant attention due to its atomically flat surface, free of dangling bonds, and excellent insulating properties suitable for nano-electronic applications [21]. For instance, h-BN serves as an ideal substrate and encapsulation layer for other 2D materials [2, 22], preserving their inherent electrical and material properties from environmental degradation. Furthermore, h-BN has been widely used as passive components in 2D electronic devices, including tunneling barriers [23] and gate insulators [24]. Additionally, a variety of active devices such as resistive switching memories [25] and RF switching mediums [26] have been recently reported. Recent studies have also explored its potential in the fields of quantum optics [27] and optoelectronics [9, 28].

However, the integration of wafer-scale ultra-thin h-BN films into next-generation nanoelectronics presents significant challenges. Since h-BN is a high-temperature phase of BN materials [29, 30], its most common large-scale synthesis method, chemical vapor deposition (CVD) requires substrate temperatures exceeding 900 °C [31]. This process is typically performed on transition metal foils [29, 31–34] and other specific substrates [35], producing films ranging from a few microns to tens of microns in size. While thermally activated CVD enables high-quality h-BN film growth, the elevated temperatures pose a major obstacle for CMOS circuit integration, as modern semiconductor chips are highly susceptible to temperatures exceeding 300 °C [36]. Additionally, scalable 2D material-based device fabrication requires transfer-free deposition techniques and low-temperature growth processes to preserve intrinsic material properties while ensuring compatibility with mass production.

Given these challenges in h-BN synthesis and integration, an alternative material with comparable insulation properties but more favorable processing conditions is required. Amorphous boron nitride (a-BN) has emerged as a promising candidate, offering similar insulating properties while enabling direct wafer-scale growth at much lower temperatures (~ 200 °C). This advantage allows for deposition on a diverse range of substrates, including metals, flexible polymers, oxides, and other 2D materials. This versatility eliminates the need for complex transfer processes and overcomes the temperature-related challenges associated with h-BN integration into semiconductor devices. Additionally, despite lacking long-range atomic order, a-BN retains essential insulation properties comparable to those of crystalline h-BN, making it an attractive candidate for nano-electronic applications [7, 15].

One of the key advantages of a-BN is its ultra-low dielectric constant (~ 2), which minimizes resistance–capacitance (RC) delays in high-speed circuits. This property is particularly crucial for next-generation nanoelectronics, where miniaturized interconnects face significant reliability challenges due to metal diffusion. Unlike traditional low-k dielectrics, a-BN offers robust mechanical and electrical properties that prevent metal diffusion into the substrate, ensuring long-term stability. [7]. Furthermore, recent studies have shown that a-BN is mechanically more flexible than h-BN due to its lower Young’s modulus, providing enhanced mechanical resilience under stress and making it particularly suitable for wearable and stretchable electronics [37].

In principle, a-BN film can be produced by various methods at much lower temperatures than its crystalline counterparts. In this context, ultra-thin a-BN films ranging from 2–17 nm in thickness were demonstrated using low-temperature pulsed laser deposition (PLD) [38]. Dielectric constant of > 6 at 1 kHz, breakdown voltage of 9 MV cm^−1^, and bandgap of 4.5 eV (comparable to that of single crystal h-BN) were reported. These uniformly grown a-BN films, possessing promising electronic properties and unique optical features such as optical transparency, render them a promising material platform for various large-scale 2D-based devices. An exceptional insulation property is realized in a-BN film grown on Si through radio frequency sputtering. Spectroscopic studies have demonstrated that these films contain sp^2^-bonded BN, with a breakdown failure and a dielectric constant of 10 MVcm^−1^ and 3, respectively [39]. Furthermore, ultra-low dielectrics are essential to minimizing signal propagation delays in electronic circuits.

In this context, a-BN film derived from a borazine precursor exhibits an ultra-low dielectric constant of approximately 2, excellent mechanical strength, and material stability [40]. It has been demonstrated that boron doping during plasma dissociation reduces the* k* value on n-type substrates. Conversely, the growth rate is a critical parameter for enhancing the mechanical strength that originates from the formation of hexagonal ring stacking frameworks in the material. Atomically thin 2D materials are subject to degradation when exposed to the external environment, which can lead to performance failures in 2D-based devices [41]. Under ambient conditions, silanol groups on the SiO_2_ surface cause localized charge accumulation at the interface between 2D materials and SiO_2_ [42]. Recent studies have shown that encapsulating sensitive 2D layers between smooth CVD-grown a-BN films not only enhance but also preserve the electronic properties of high-quality 2D layers in air conditions over time [15, 43].

Beyond electronics, a-BN coatings can be used in coatings applications such as a solution for high temperature mold release, protection layer for a variety of surfaces including metals, ceramics, composites, etc. [44–46]. a-BN-based coatings are chemically stable and resistant to harsh environmental conditions, such as high humidity, moisture and other similar aggressive settings. Unlike many other materials, a-BN is non-toxic, which makes it suitable to implement for applications that involve direct contact with humans, thus, the biocompatibility of a-BN makes it a promising platform for medical applications [47].

This paper aims to review the state-of-the-art developments in the growth and properties of a-BN, and its applications in emerging nanoelectronics and photonics. We focus on the most recent experimental findings, including the material properties of a-BN, growth techniques, and their associated electrical and optical properties, and how these features contribute to a wide spectrum of applications, from passive components to active devices and beyond. Section 2 will discuss newly explored electrical and optical properties and strategies for their manipulation and enhancement. Subsequently, the latest advancements in a-BN-based devices are presented, with an emphasis on resistive switching devices, encapsulation layers, gate insulators for FETs, effective diffusion barriers, and photon emitters and detectors for next-generation nanoelectronics and photonics.

## Properties

### Structural properties and characterization

**Fig. 1:**
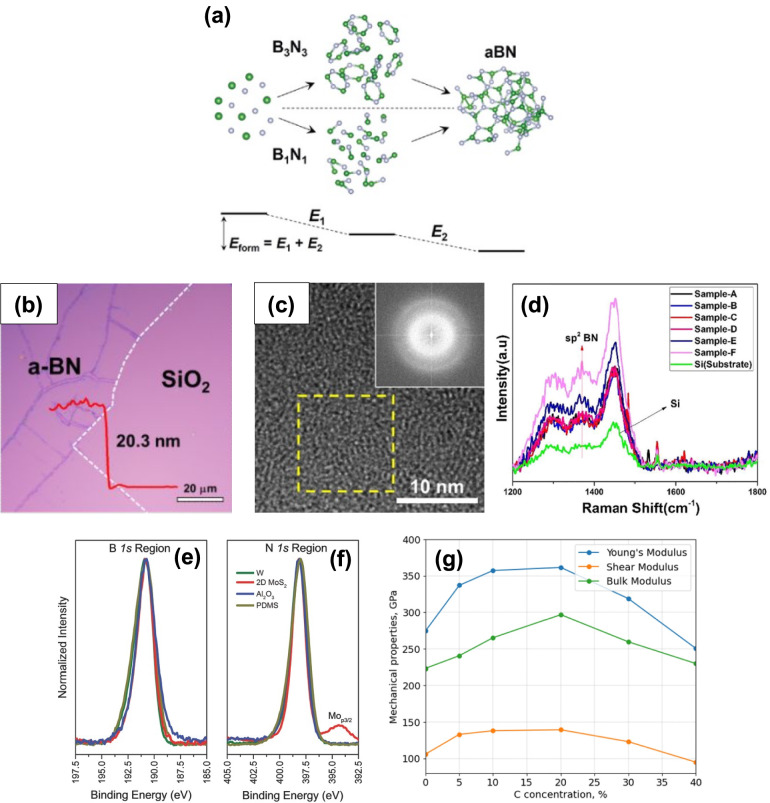
**a** Schematic illustration of forming a-BN through ring (R) and dimer (D) mechanisms. Reprinted from [40]. **b** OM image of as-grown a-BN thin film on SiO_2_ with a thickness of 20 nm. Reprinted from [15]. **c** High-resolution TEM image of a-BN demonstrating the absence of atomic order in the structure. The inset displays the magnified image and the diffraction pattern, respectively. Reprinted from [15]. **d** Raman spectra of a-BN thin films with varying thicknesses. Reprinted from [39]. **e** XPS B1 s and (f) N1 s regions of a-BN films grown at < 200 °C on various representative substrates. Reprinted from [38]. **g** Bulk, Young’s, and shear moduli of a-BN as functions of C-doping. Reprinted from [37]

An ultralow *k* a-BN film was reported to be grown by plasma chemical vapor deposition (ICP-CVD) and low-pressure chemical vapor deposition (LP-CVD) on n^++^-Si and SiO_2_ substrates at lower temperatures using Borazine (B_3_N_3_H_6_) as a precursor [14, 15, 48, 49]. Precursor polarity and growth rate are two critical parameters for synthesizing high-quality, low-k, and dense a-BN films on 300 mm Si wafers. An initial computational study [40] described the growth process of experimentally prepared borazine-a-BN films (e.g., *k* ~ 2.11, density ~ 2.1 g cm^–3^, and intrinsic Young’s modulus > 50 GPa) on Si wafers at 400 °C focusing on two elemental processes related to precursor polarities (Fig. 1(a)) [50, 51]. The a-BN film remains transparent if grown at sufficiently low temperatures (e.g., before crystalline sites begin forming in the film). Figure 1(b) illustrates the transparent a-BN film on a SiO_2_ substrate at 250˚C [15] where it is nearly impossible to detect any color variation between the SiO_2_ and a-BN/SiO_2_ films. In a-BN, boron and nitrogen atoms are covalently bonded, and the broad Raman peak before and after the growth of a-BN on SiO_2_ remained consistent [15]. The tunneling electron microscope (TEM) images show that a-BN films are amorphous, lacking long-range atomic order (see Fig. 1(c)). Furthermore, angle-dependent near-edge X-ray absorption fine structure (NEXAFS) analyses allow for deeper insight into the chemical and electronic structure of a-BN. NEXAFS spectra of the a-BN sample demonstrate that the resonance at 192 eV arises from the 1 s → π* transition in boron [52]. The negligible variation with the X-ray incident angle underscores the random distribution of BN planes throughout the material. NEXAFS also reveals the sp^2^-hybridized nature of grown a-BN films [52, 53]. Raman spectra show the presence of sp^2^-boron nitride phases (see Fig. 1d). Peaks at 1450 cm^−1^ represent the third-order transverse optical (TO) phonon from the Si substrate, and vibrations from sp^2^-bonded modes appear at approximately 1370 cm^−1^ [54]. As indicated in Fig. 1 (e)-(f), the B/N atomic ratio is approximately 1:1.08 with B1 s and N1 s peaks at 190.4 eV and 397.9 eV, respectively, confirming the films are sp^2^-bonded B and N [55]. Note that the role of doping elements, including carbon and hydrogen, significantly impacts the structural properties such as thermal and mechanical stability. Additionally, how the sp^3^/sp^1^ and sp^3^/sp^2^ ratios influence highly disordered a-BN structures is critical [56]. For instance, at a certain hydrogen doping level, the concentration of sp^3^-hybridized atoms peaks, resulting in a 20% improvement in thermal stability and mechanical properties [57]. Results indicated that hydrogen-doped a-BN (a-BN: H) samples became more stable until the H atoms concentration reached 8%, due to an increase in the sp^3^/sp^1^-hybridized atoms ratio. Additionally, simulation results showed that increasing carbon doping up to 20% enhances the sp^3^ fraction, thereby improving thermal and mechanical stability (as illustrated in Fig. 1. g). Here, higher carbon content and C–C bonds in a-BN samples lead to the graphitization of films, corresponding to lower sp^3^ hybridization levels [37], demonstrating a correlation between these structural features and thermal stability.

### Optical properties

Boron nitride has attracted significant interest as an active optical material, serving both as a photon source and detector, particularly in ultraviolet emission spectral ranges [58–61]. Strong coupling of exciton-phonon interactions and the presence of boron and nitrogen vacancies create stable mid-gap states, leading to efficient single-photon emitters (SPEs) in both visible and UV spectra [62, 63]. These localized vacancies are extremely stable as they form during crystal growth and annealing, resulting in robust SPE performance. BN-based SPEs demonstrate controllable polarization and emission with high quantum efficiencies, as the emitting region is very close to the film's surface [64]. In h-BN, as an ultra-wide bandgap material, these defects are expected to be optically active with ground and excited states. Thus, effective defect engineering enables wide spectrum emission, suitable for advanced applications such as BN-based optical quantum computing.

However, a-BN has also shown potential for SPE applications due to its sensitivity to defects, but fundamental reports on its optical properties are still limited. Boron nitride, whether in monolayer, few-layer h-BN, or a-BN, exhibits strong sensitivity to defects and vacancies, which play a key role in creating localized electronic states that trap charge carriers and facilitate quantum localization. For instance, in monolayer h-BN, vacancy-induced states create localized electronic states that can trap charge carriers, significantly altering the material’s electronic properties. These localized vacancies can couple with neutral excitons (X⁰), forming defect-bound excitons, which are essential for single-photon emission (SPE). The coupling of excitons with defect states leads to the formation of stable localized states, and upon recombination, these defect-bound excitons emit photons at discrete wavelengths, a key feature for SPE. Though SPE phenomena in a-BN have not been widely reported, there remains significant potential for vacancy-induced or doping-induced SPE in a-BN, suggesting that further investigation into defect engineering in a-BN could open new possibilities for single-photon emission.

**Fig. 2:**
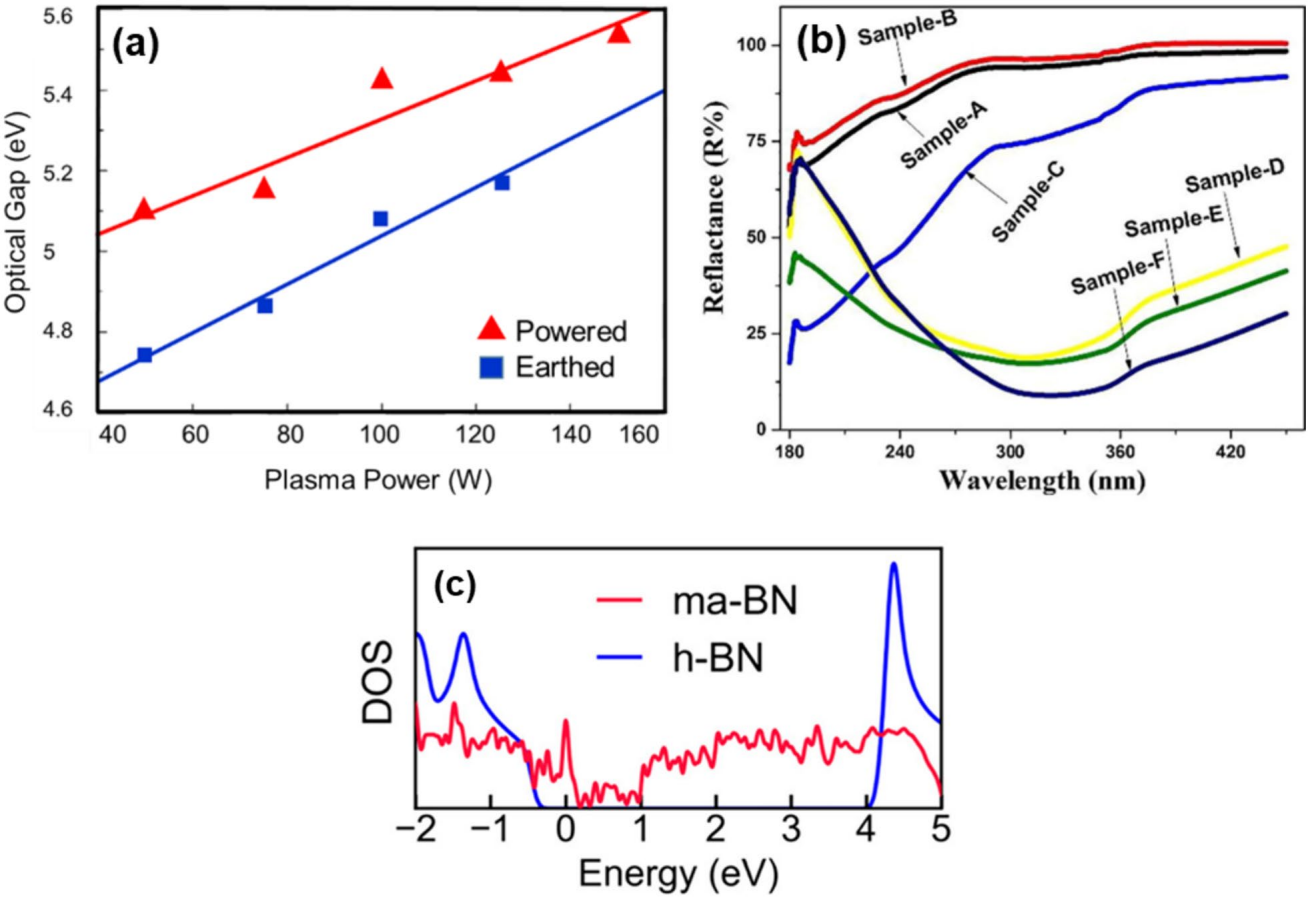
**a** The optical gap of a-BN film. Reprinted from [64]. **b** The reflectance of a-BN films with varying thicknesses. Reprinted from [39]. **c** Calculated DOS of monolayer a-BN (ma-BN) and h-BN, illustrating the continuum of defect states within the bandgap. Reprinted from [16]

One of the early studies on the optical properties of PE-CVD-grown a-BN films was conducted by *Mendez* and *Muhl*, where they demonstrated transmittance, refractive index, and optical bandgap [64]. They analyzed the impact of growth parameters on these properties. For example, the optical bandgap widened following bombarding due to the removal of defects within the gap (see Fig. 2(a)). The refractive index of the films on silicon deteriorated as the pressure increased. UV reflectance varies with the thickness of the a-BN thin film, as shown in Fig. 2 (b). Random and relatively broad peaks corresponding to different film thicknesses (samples A to F with thicknesses of 12.8, 20.4, 34.3, 40.6, 50.4, 79.9 nm, respectively) indicate that the grown film lacks any crystal format [39]. The graph reveals that thinner a-BN films exhibit higher UV reflectance across the wavelengths. For example, the film approximately 13 nm thick (sample A) displayed nearly 100% reflectance, whereas the thicker film (sample F, 80 nm thick) showed a peak reflectance of less than 50%. It is noteworthy that low-temperature grown a-BN films are transparent [65–67], which prevents the oxidation of 2D materials grown on an a-BN substrate and enables transfer-free fabrication of 2D transistors on flexible substrates. Moreover, transparent layers are useful in a wide range of applications, including flexible electronics [68–70], and others [65, 71–74]. Ultimately, most commercial PDs require wafer-scale materials, a challenge given the current h-BN growth approach. The size of the h-BN films is restricted to less than 1 mm under low-pressure and high-temperature conditions [75]. Consequently, a low-temperature, transfer-free method is highly desirable for the mass production of high-quality BN films for LEDs [76]. It is noteworthy that transparent a-BN films may have significant potential for optical and optoelectronic device applications. From this perspective, it is crucial to adequately consider the correlation between synthesis parameters such as temperature and film thickness with transparency.

### Electrical properties

Recently, a-BN has emerged as a promising insulator with a high bandgap, high breakdown voltage, and ultra-low dielectric constant. Material characterization analyses have shown that a-BN possesses a very smooth surface morphology, is free of charged impurities and dangling bonds, and remains stable at high temperatures. From this perspective, a-BN is becoming a key platform in emerging electronic applications, including resistive switching memories (RSMs), RF switches, and FET devices. Unlike h-BN, extensive research into the electrical/electronic properties of a-BN is lacking. Theoretical studies indicate that the electronic density of states (DOS) of monolayer a-BN (ma-BN) differs from its hexagonal counterpart by having a wider bandgap. ma-BN exhibits a continuous spectrum of in-gap states [16], which complicates the doping process, although h-BN can be doped as n-type or p-type (see Fig. 2(c)). Both experimental [14, 15] and theoretical [16, 77] studies confirm that a-BN lacks long-range atomic order, affirming its amorphous nature. Additionally, the presence of many distorted noncanonical hexagons and other polygons in the ma-BN structure has been demonstrated. Note that the in-gap states of DOS for bulk a-BN (thicker than one atomic layer) may differ from ma-BN. Thus, a thorough theoretical and experimental study is needed to determine the relationship between thickness and DOS spectrum properties. Studies have shown that the current level in sulfur-doped a-BN increases nearly two orders of magnitude after annealing [5]. The electrical breakdown field for the a-BN film with a thickness of 3 nm was observed at approximately 7.3 MVcm^−1^, almost double that of h-BN. Compared to other insulating materials with *k* < 2, it boasts the highest reported breakdown field [7]. Furthermore, the a-BN film exhibited extremely low leakage current density and ultra-low *k* values, highlighting its potential for use in 3-nm-node nanoelectronics. It is noted that the fundamental electronic properties of a-BN layers can vary significantly depending on growth and fabrication techniques. For instance, the breakdown field and dielectric constant are heavily dependent on film quality and the density of defects and pinholes. CVD-grown a-BN tends to have a smaller dielectric constant compared to other methods. In well-known h-BN films, various factors significantly impact the electrical properties. The symmetry of layer stacking [78] can alter the electronic bandgap (*E*_g_), potentially changing an insulating BN into a conducting material [79]. It has been observed that edge termination can modify the electronic structure, transforming an insulator into a semi-metallic material [80]. Moreover, impurities, vacancies, adatoms, and strain significantly influence the electrical properties of BN [81]. However, the effects of these factors on the electrical properties of a-BN have not yet been fully elucidated. Research indicates that the growth temperature can alter the absorbance (and thus, the bandgap) and the relative dielectric constant of an a-BN film [82]. The higher the growth temperature, the lower the dielectric constant of a-BN samples. For example, a dielectric constant of approximately 4.3 at 250 ˚C (*E*_g_ of 5.7 eV) increased to 8.6 (*E*_g_ of 5.8 eV) at 65 ˚C, where the dielectric breakdown was 4.4 MV/cm at 65 ˚C and increased to 8.2 MV/cm at 250 ˚C. This evolution shows promise for electronic applications. As the physical thickness of gate dielectrics is scaled down, ultra-low power 2D FETs require a high dielectric constant to minimize leakage current.

## Synthesis of a-BN: challenges and opportunities

**Fig. 3:**
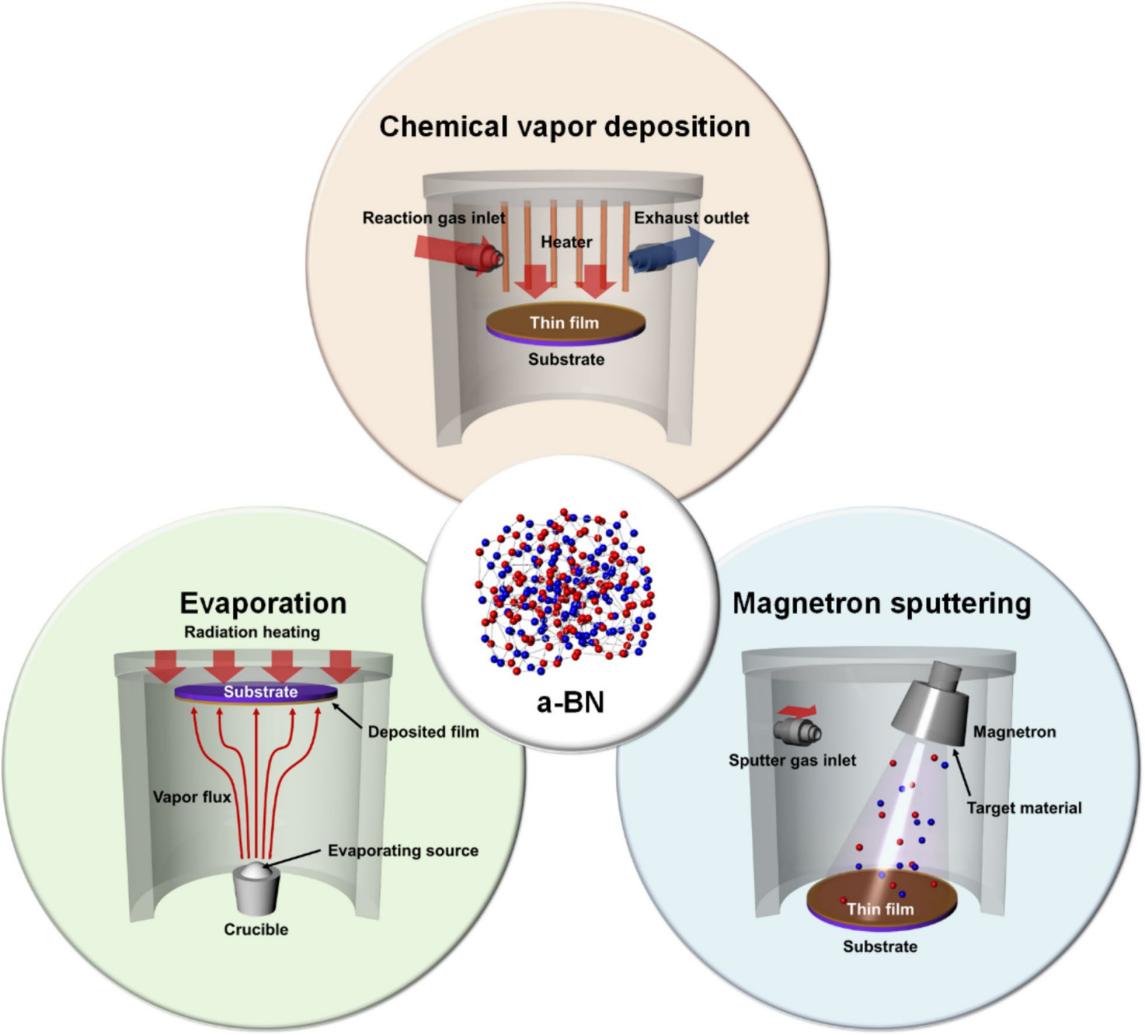
Schematic illustration for the most common synthesis methods of a-BN, including sputtering, chemical vapor deposition (CVD), and evaporation

In the quest for novel materials with tailored properties, researchers have explored integrating amorphous boron nitrides into two-dimensional (2D) materials using various techniques. Generally, synthesis methods for 2D materials rely on mechanical and liquid exfoliation (top-down growth) or vapor phase transport (bottom-up growth) [83]. This chapter investigates the synthesis methods used to develop amorphous boron nitrides within 2D materials, discussing typical methods such as chemical vapor deposition (CVD), plasma-assisted CVD (PACVD), ion implantation, and chemical reactions. Figure 3 presents the most common synthesis methods for a-BN discussed in this section.

### Chemical synthesis methods

Chemical methods for synthesizing boron nitrides are generally conducted under a vacuum and typically involve reactions between boron-containing compounds and nitrogen sources. In contrast to top-down methodologies, chemical synthesis offers significant advantages in the fabrication of large-area boron nitrides. This review details prevalent chemical methods used to synthesize boron nitrides, with a particular emphasis on a-BNs.

#### Chemical vapor deposition (CVD)

**Fig. 4:**
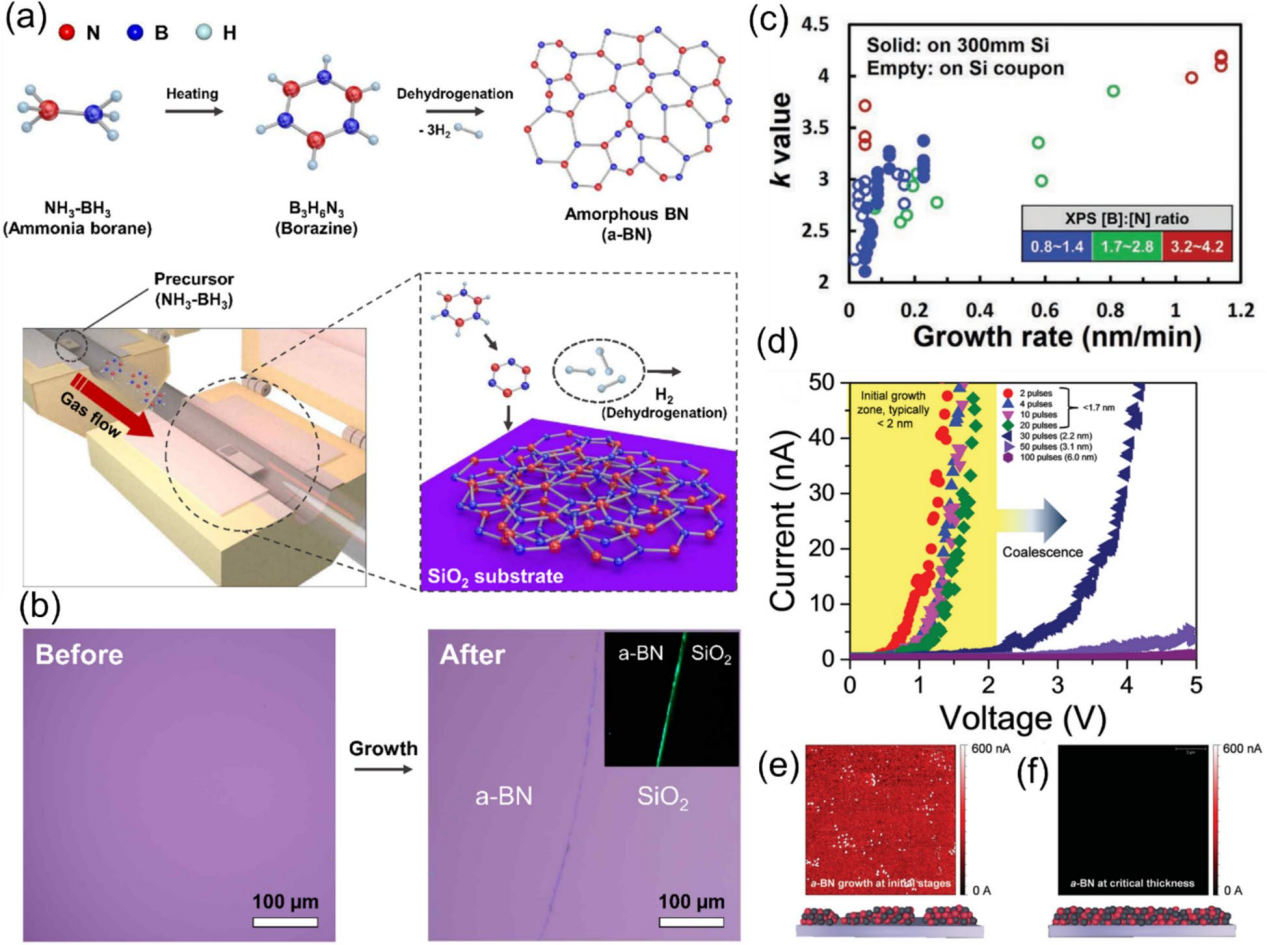
**a** Schematic of the growth mechanism of the a-BN film. **b** Optical microscopic images of 300 nm SiO_2_/Si substrate before and after a-BN growth. Reprinted from [15]. **c** *k* values of a-BN thin films (extracted from the slope of equivalent oxide thickness versus a-BN film thickness) grown on Si coupons and 300 mm Si wafers at various growth rates and [B]: [N] ratios. Reprinted from [40]. **d** I–V characteristics of a-BN and corresponding number of laser pulses conducted by C-AFM. Related C-AFM image at (**e**) initial growth stage at 2 pulses, and (**f**) at the critical thickness of 20 pulses. Reprinted from [38]

CVD is a widely employed technique for the synthesis of h-BN [49, 54] and a-BN [15]. In the CVD process, a precursor gas containing boron and nitrogen is introduced into a vacuum system, where it reacts on the substrate to form amorphous boron nitride. By adjusting the flow rate and partial pressure of each precursor, one can precisely control the number of molecules involved in the process, thereby controlling the amorphous structure and chemical composition of a-BN. The nucleation and growth of 2D materials, including a-BN, through the CVD process, depend on several parameters such as substrate type, process temperature, working pressure, and gas flow rate. Another crucial factor in determining the uniformity and chemical composition of the CVD synthesized films is temperature. In principle, the process temperature can influence the kinetics of the reaction and precursor reaction in the gas phase, thereby affecting the deposition rate at the substrate level. Normally, the precursor molecules can obtain the energy required to overcome the activation energy barrier, which in turn facilitates the reaction and promotes the formation of a-BN. Increasing the temperature can enhance the quality of a-BN films; however, excessive heat might alter the chemical composition and reduce uniformity. For example, the successful growth of a-BN films through the CVD process was reported at 250 °C and a working pressure of 110 Torr [15]. Moreover, different substrates can be combined to create heterostructures ideal for the growth of complex 2D materials. The substrate on which a-BN is grown can play a crucial role by influencing the nucleation, growth, and surface quality of the film. Thus, selecting an appropriate substrate is essential to ensure initial growth stages (growth of the initial few layers), good adhesion, low stress, and minimal reactions at the interface. Typical substrates used in the CVD process to develop a-BN include Si and SiO_2_ wafers, and metal foils such as Cu, Ni, and Al_2_O_3_. Some researchers have also reported the growth of h-BNs on graphene/Ge substrates [84]. Representative centimeter-scale a-BN films were synthesized using a double-zone LP-CVD system, which includes two zones: a precursor-vaporization zone (furnace 1) and a reaction zone (furnace 2) (see Fig. 4(a)). An ammonia-borane complex (NH_3_-BH_3_) powder and various substrates of centimeter size including graphene, Ge, Si, and SiO_2_ were used for growth. Dehydrogenation of B_3_N_6_H_3_ molecules was carried out by heating furnace 2 to 250 °C, under 110 Torr pressure with flowing H_2_, and B_3_N_6_H_3_ molecules were deposited on the substrate in furnace 2. Figure 4(b) displays optical images of a 300 nm SiO_2_/Si substrate before (left) and after (right) a-BN growth, illustrating that the as-grown a-BN film is transparent and uniform. It is important to note that post-growth annealing at high temperatures and the growth temperature of the substrate can influence the color and transparency of the a-BN film. As crystallization occurs at higher temperatures, the optical band gap decreases, leading to increased light absorption and changes in the refractive index, which together influence the color and transparency of the a-BN film. However, the impact of temperature on the chemical composition, film uniformity, and the electrical and optical properties requires further exploration.

The CVD process primarily employs thermal energy for depositing films. When coupled with plasma, the process is referred to as plasma-enhanced CVD (PECVD) or plasma-assisted CVD (PACVD), which are well-established methods for synthesizing thin films. The PECVD process utilizes plasma-driven energy in addition to thermal energy to enhance the chemical reaction and lower the necessary substrate temperature. Thus, it allows a wider variety of materials to be processed, particularly those sensitive to high temperatures. Recently, mass production of ultralow-k a-BN was achieved using the PECVD system at a process temperature of 400 °C on a 300 mm Si wafer [40]. The *k* evolution of a-BN films with varying growth rates is illustrated in Fig. 4 (c). a-BN thin films (~ 2.5 nm-thick) with a low-*k* of 2.11 and a high density of 2.1 g cm^−3^ were achieved. The higher the growth rate, the higher the *k* values, where films grown on Si coupons exhibited relatively large *k* values, especially at higher growth rates (see Fig. 4(c)). Similar outcomes were obtained using an atomic layer deposition (ALD) method at a low growth temperature of 100 °C [85].

#### Atomic layer deposition

Atomic layer deposition (ALD) is a CVD-based technology for thin film growth. This method has garnered significant interest in semiconductor and MEMS applications due to its defect-free structure, uniform thickness, and excellent conformity of the ALD coatings. To initiate the process, a precursor gas is introduced into a vacuum chamber where the gas molecules adhere to the substrate as a monolayer and undergo chemisorption. Subsequently, the remaining gas molecules are evacuated and a different precursor gas is introduced. This leads to chemisorption of gas molecules on the initially deposited layer, forming the first monolayer. This sequence is repeated to achieve the desired film thickness. The coating thickness achieved by ALD can be precisely controlled down to the Angstrom level [86, 87].

To deposit a-BN films by ALD, a substrate must be sequentially exposed to boron and nitrogen-containing precursors cyclically. It is important to note that the choice of precursor and deposition condition significantly influences the properties of the grown a-BN films. For example, Wolf et al. reported the ALD synthesis of conformal and pinhole-free a-BN films using N_2_H_4_ and BCl_3_ precursor gases [87]. Synthesis of h-BN using the ALD method, described by authors as a hydrogen diffusion barrier, was also reported [90]. However, the diffusion of atomic hydrogen depends on the h-BN sheet orientation. Moreover, ALD-grown a-BN films showed that deposition temperature critically affects film components such as B-N, B-O, N-B, and N–H, as well as B-O [80]. A higher density of B-O XPS peaks was observed at lower temperatures, suggesting the incorporation of O atoms into the thin films. Additionally, an increased concentration of N–H was noted at lower growth temperatures. Since oxidation is elevated at lower temperatures, a greater defect concentration in the film may alter leakage current and dielectric breakdown fields.

#### Pulsed laser deposition (PLD)

Pulsed laser deposition (PLD) involves striking a target material with a high-intensity pulsed laser beam in a chamber under high vacuum conditions. The laser beam is absorbed by the target, emitting energetic particles toward the substrate material in a plasma plume. Key process parameters such as chamber pressure, substrate temperature, and laser settings including energy density and pulse repetition can significantly affect the deposited layer. Another advantage of PLD is that the target stoichiometry is effectively preserved in the deposited film due to confined beam absorption volume in a non-equilibrium process [89, 90]. PLD has also been used to grow a-BN films from BN targets. Wang, et al., produced a-BN films using a 300-femtosecond laser with a pulse energy of ~ 1 mJ, resulting in uniform growth, mid-infrared transparency, and excellent scratch-resistance [90]. PLD has also enabled the deposition of a-BN films at relatively lower temperatures, producing a higher band-gap than CVD-grown h-BN films [38]. The improvement is attributable to superior roughness, stoichiometry, and a defect-free structure. The I-V characteristics of a-BN films obtained using a conducting atomic force microscope (C-AFM), as depicted in Fig. 4(d)-(f) [39]. Pinholes were observed in the film body before reaching 20 pulses, with minimal changes in I-V behavior. The breakdown voltage significantly increases once complete surface coverage of the a-BN thin film is achieved (~ 1.67 nm-thick layer). As illustrated in Fig. 4(d), film breakdown voltages increase at higher pulse lasers. This dependency on the number of laser pulses is linear during uniform growth mode. The dielectric breakdown field rose from 5 to 9.8 ± 1.0 MV cm^−1^ in the transition from nearly formed films to fully coalesced 2.2 and 3.1 nm samples (see Fig. 4(e)-(f)), where the electronic behavior follows the Fowler–Nordheim tunneling theory.

#### Ion implantation

Ion implantation involves the direct introduction of boron and nitrogen ions into a 2D material's lattice, and nitrogen ions can also be implanted into boron-deposited films. The implanted ions disrupt the crystalline structure, creating amorphous regions within the 2D material. This method provides precise control over the location and density of these amorphous regions, making it ideal for creating customized structures. Parameters such as implant voltage and ion dose are crucial in the formation of the BN microstructure. It has been reported that BN films deposited by ion implantation [91] exhibit excellent adhesion to the substrate [92]. Also, it was found that a high N/B compositional ratio facilitates crystallization of BN film. On the other hand, ion implantation is used to modify the crystalline structure of h-BN films. Lisema et al. have implemented ion implantation to introduce defects into the CVD-grown BN nanotubes selectively. The Raman spectra showed that applying ion flounce to the samples generates an amorphous h-BN peak [93], where the Ag ion implantation into the hollow BN nanoparticles led to the transformations of the crystalline BN nanotubes to the amorphous phase [91, 94].

### Physical synthesis methods

Physical vapor deposition (PVD) is a term that encompasses a variety of deposition processes in which a solid material is transformed into the vapor phase through a physical mechanism and then condenses onto a substrate as a thin film. The vaporization can be driven by different mechanisms, including electron gun, resistance heating, or energetic ion bombardment, leading to various PVD modes. Currently, several PVD techniques are widely used in industry and mass production, particularly sputtering and evaporation, which have gained significant attention in nanofabrication and semiconductor sectors. Compared to chemical synthesis methods, PVD techniques are less commonly used for synthesizing boron nitrides, especially in their amorphous form. Despite limited use, a concise overview of PVD techniques used for the synthesis of amorphous boron nitrides is provided.

#### Sputtering

The term'sputtering'refers to several processes encompassing the same physical phenomena. Notably, magnetron, cathodic, ion-beam, reactive, and non-reactive sputtering are employed. The principle of sputtering involves the ejection of source material atoms or molecules through bombardment by energetic particles and their deposition onto the substrate surface. In sputtering, initially, a sufficient number of high-energy ions must collide with the target surface to dislodge atoms from their positions. These atoms are then accelerated towards the substrate, ideally with minimal collisions with gas molecules. Therefore, a vacuum system that maintains low pressure (< 1 Pa) and correspondingly a large mean free path (MFP) is essential. Finally, the dislodged atoms deposit on the substrate surface, forming a thin film.

Among all sputtering techniques, magnetron sputtering is widely used to deposit high-quality thin films. Depending on the desired film properties, the cathode can be connected to direct current (DC), radio-frequency (RF), pulsed-DC (P-DC), or high-power impulse magnetron sputtering (HiPIMS) power supplies. The sputtering technique is also employed in the synthesis of boron nitride. Recently, several groups have reported the development of a-BN films on sapphire substrates using the radio frequency sputtering method [39]. Studies on electrical properties showed that the breakdown failure of sputtered a-BN films was greater than 10 MV/cm, while the dielectric constant exceeded 3. The growth mechanism of a-BN through sputtering involves two main thin film evolution stages, which are somewhat influenced by the number of laser pulses: nucleation and thin film formation.

#### Evaporation

This type of PVD utilizes a deposition process via thermal means, commonly involving techniques such as electron beam (e-beam) evaporation and resistive thermal evaporation. During e-beam evaporation, an electron gun with a hot filament propels high-energy electrons toward the crucible containing the material for coating through thermionic emission. The kinetic energy of the e-beam converts to heat, resulting in evaporation of the source material. Resistive thermal evaporation employs a simpler method, where the material is heated to its evaporation point under high vacuum. Continuous particle production is maintained by a mechanism that feeds wire to the hot boat. E-beam evaporation was used to synthesize materials like amorphous boron carbide(a-BC) [94]. There are no reports of using evaporation as a common technique for depositing a-BN films. However, given the growth similarities between a-BC and a-BN, e-beam evaporation has potential for producing high-quality a-BN films in the future. In a rare instance, an amorphous phase was observed on the Si substrate in HR-TEM images, although the goal was to develop a cubic phase [95]. This amorphous phase was attributed to the rapid cooling of films, and the disparity in lattice parameters as well as stress-related phenomena (resulting from film cooling) might have facilitated the amorphous BN phase formation using a similar E-beam technique. Factors such as scalability, film quality (e.g., surface morphology, contamination-free films, etc.), growth condition, transfer process, and growth temperature stand as critical ahead of commercializing a-BN.

Taking all factors into account, CVD emerges as a promising method for scalable and CMOS-compatible production of a-BN materials, making it a pioneering approach for device applications. The demand in 2D-material-based nanoelectronics for transfer-free approaches and low-temperature growth conditions is crucial to preserving material quality and enabling large-scale production [94–96]. Direct growth on the host substrate addresses transfer-related challenges, such as organic residuals at the 2D/a-BN interface, which are major obstacles to achieving optimal device performance. Recent advancements in direct CVD growth on various commercial substrates, including SiO_2_, Si, and graphene [15], show promise in overcoming these hurdles. However, high-temperature processing remains a limitation, particularly for integrating sensitive 2D semiconductors.

Alternatively, PLD enables lower-temperature deposition of a-BN films while offering higher breakdown strength and a larger bandgap compared to CVD-grown h-BN films. However, film quality remains a challenge and depends on factors such as laser pulse count and growth temperature. Optimizing these parameters is essential to achieving consistent film properties. On the other hand, ALD-grown a-BN films, while advantageous for precise thickness control and uniformity, suffer from higher defect concentrations and oxidation issues, especially at low growth temperatures. These defects impact dielectric properties and stability, necessitating further refinement. Ultimately, each synthesis method presents distinct advantages and challenges, with film quality, electrical properties, and large-scale fabrication feasibility being key factors that require further investigation.

## Applications

**Fig. 5:**
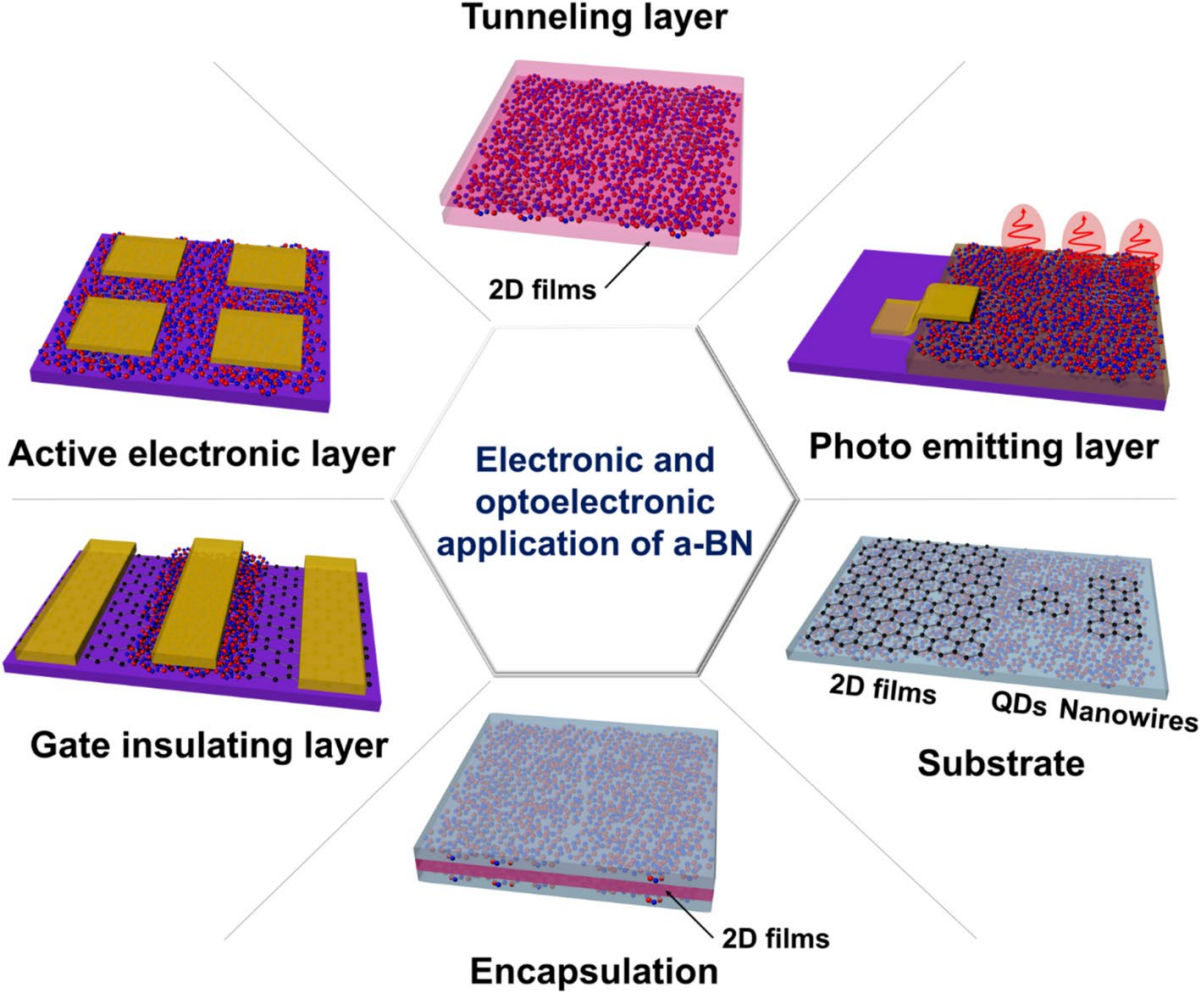
Schematic illustration of current and potential applications of a-BN in nano-electronic and optoelectronic devices

This section provides a comprehensive overview of the diverse applications of a-BN across various technological domains including electronic and optoelectronic and underscores the growing interest in a-BN as a multifunctional material with promising roles in next-generation device engineering (Fig. 5).

### Electronic

a-BN and h-BN possess robust physical properties [96–101], high thermal stability [99, 101–103], and a stable chemical composition that enables their use as an active layer, thereby stabilizing and enhancing the electronic performance of ultra-thin 2D electronic devices [104–107]. The dangling-free, atomically smooth surfaces, and relatively small lattice mismatches between the active 2D layers and substrates facilitate sharp and clean interfaces with 2D materials, thus improving device performance. For example, properties such as bandgap [108, 109], mobility [110, 111], and Fermi velocity [112, 113] of 2D layers can be tailored using BN substrates. This characteristic establishes BN materials as strong candidates for substrates in ultra-thin 2D layers. Additionally, a-BN and its crystalline counterpart are promising candidates for various electronic components like memories [114], FETs [115], and neuromorphic devices [116]. On the other hand, inherent defects and impurities in BN materials (particularly h-BN and potentially a-BN) spawn diverse research directions in nano-photonics [1, 15, 23, 26, 38, 40]. However, further research is required to thoroughly understand the defect profile and control mechanisms in both monolayer and bulk a-BN films before these materials can be employed in practical applications.

#### Memory devices with synaptic functionality

**Fig. 6:**
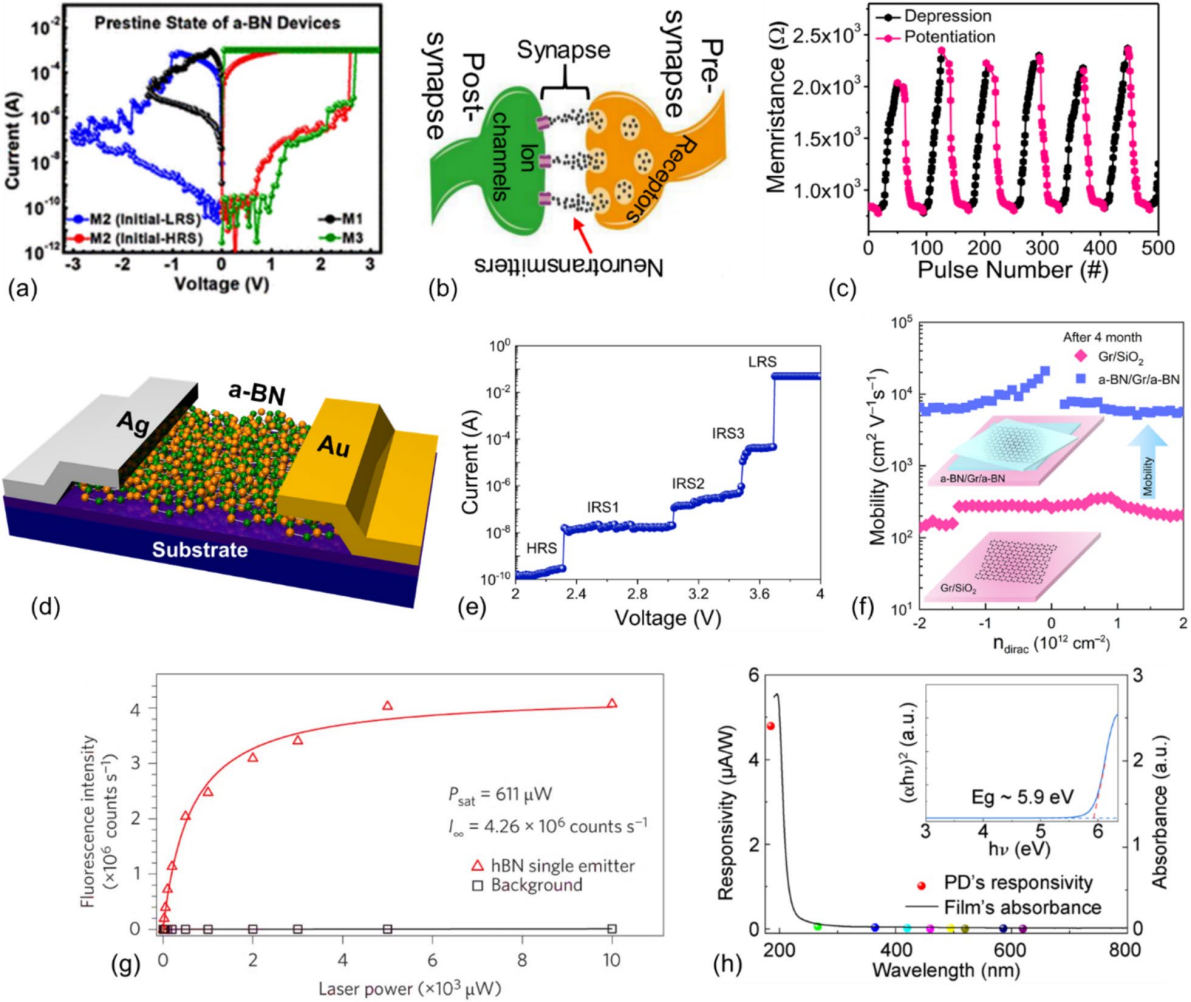
**a** Resistive switching in a-BN thin films, reprinted from [120],** b** schematic illustration of a pre-and postsynaptic neural system mimicked by a-BN memory devices. **c** differentiation and potentiation as functions of pulse number for a-BN memory, reprinted from [132], **d** schematics of multilevel aBN nonvolatile memory, **e** I-V characteristics of memory device shows quinary switching resistance including three intermediate states (IRS1-IRS3) [133], **f** field-effect mobility for exposed and sandwiched 2D-FET between two a-BN films, as prepared and after 4 months, reprinted from [15]. **g** Fluorescence curve recorded from a single defect in h-BN film. Reprinted from [163]. **h** Responsivity (at different wavelengths) and absorption characteristics of the BN photodetector with an inset showing the bandgap estimation, reprinted from [168]

Amorphous Boron nitride, characterized by its disordered atomic structures and defects, exhibits properties that significantly differ from those of its crystalline counterparts. Despite the exceptional functionality of h-BN-based memory devices, the primary challenge for BN-based memory integration is the high growth temperature required (∼800 − 1050 °C) [117]. An a-BN can be deposited at temperatures below 250 °C using CVD and PLD, thereby overcoming the temperature barrier for integrating a-BN devices into CMOS technology [15, 118]. Consequently, a-BN layers produced by various synthesis methods have been used to fabricate resistive switching memory devices. Jeon et al. demonstrated a bipolar conductive bridge random access memory (CBRAM) structured with metal/a-BN/metal stacking [117]. They explained the correlation between metal-ion diffusion/migration and a-BN film thickness, and its impact on resistive switching performance. The a-BN thin films were created using sputtering deposition where the resistive states and I_ON_/I_OFF_ ratios depended on the thin film thickness (refer to Fig. 6 (a)). This dependency was attributed to the amount of in situ Ag diffusion during the electrode deposition process (the thinner the layer, the greater the diffusion). The stability of switching behavior, including endurance, retention, and sensitivity to environmental factors like humidity, varies with a-BN thickness. The greater the thickness of the a-BN film, the larger the I_ON_/I_OFF_ ratios and the higher the forming voltage, which results from the modulation of Ag diffusion by the a-BN thickness in the device. Memristive devices and flexible memories hold promise for enabling neuromorphic computing systems [22, 119–122] by mimicking synaptic behaviors. Notably, a semiconductor brain was developed by integrating memristive devices into a CMOS chip [123]. An a-BN layer sandwiched between metal electrodes can serve as a transport layer while the electrodes function as input and output terminals. The metal/a-BN/metal memristive device can then emulate synaptic-weight modulation behavior. In this context, a resistive switching device with synaptic behavior was developed using a Ti/a-BN/Si device for neuromorphic applications [124]. A change in resistance level, due to boron vacancies-assisted Ti cation diffusion involved in the conducting path, resulted in enhanced endurance and multilevel switching quality, which was characterized using temperature dependence and modeling. Additionally, potentiation, depression, and spike-timing-dependent plasticity (STDP) behavior of the Ti/a-BN/Si device is reported. The multiple conductance states demonstrated in this study could potentially enhance pattern recognition accuracy in neural network simulations for neuromorphic applications. Additionally, multi-valued memories are crucial for storing high-density information across various device structures, including NDR devices [125, 126] and RSMs [127, 128]. Memory storage can be achieved through multilevel resistive switching (MRS) devices that have a capacity exceeding one bit. Oxygen vacancies or metal ions are key in generating these multiple resistance states by forming conductive filaments (CFs) [129, 130]. RSMs have been identified as promising platforms for implementing spike-timing dependent plasticity (STDP) based on Hebbian learning rules [131]. In this context, a high-density memory configuration was introduced by Khot et al., who developed a complementary CMOS-compatible Ag/a-BN/Pt memory for synaptic device applications [130]. A schematic representation of the biological synapse junction, positioned between the pre-and postsynaptic neuron, is shown in Fig. 6(b). The a-BN memory device successfully emulated the synaptic-weight modulation effect under a sequence of potentiation and depression pulses (Fig. 6c). The device behavior gradually increased and decreased during the potentiation and depression cycles, confirming that the a-BN memory device can replicate the fundamental biological synaptic process. Furthermore, the device demonstrated significant retention time and switching reliability. The switching mechanism was attributed to the formation of a filament within the a-BN thin film. Recently, Sattari-Esfahlan et al. [131] demonstrated the multi-state characteristic, with a CVD-grown and transfer-free amorphous boron nitride film memristor that potentially rivals the logic capacity of multiple field-effect transistors. Their a-BN memory shows quinary resistive switching with five distinct resistive states (Fig. 6d-e). The formation of conductive filaments (CFs) is the primary mechanism behind the multilevel switching observed in the a-BN-based MIM structure. Boron (B) vacancies, which have a lower formation energy than nitrogen (N) vacancies, act as trapping sites for Ag ions, leading to the formation of CFs. The appearance of each intermediate state at distinct voltages results from the initiation, modification, or alteration of CF dimensions, which in turn changes the channel resistance. Furthermore, the device exhibits an exceptional On/Off ratio of ~ 10^8^ with long-term stable performance. Results demonstrate that the number of intermediate states depends on the a-BN thickness, which is possibly raised from the evolution of filament dimensions within the channel. This approach can address the common production and transfer bottlenecks associated with 2D materials and is a pivotal step toward addressing temperature incompatibility.

#### Electronic interconnect and protection layer

Capacitance and resistance delays are significant challenges limiting data processing speed [134, 135]. According to the International Technology Roadmap for Semiconductors (ITRS), the development of low-*k* dielectrics with values under 2 will be essential by 2028 to produce interconnect isolation materials that effectively act as diffusion barriers against metal migration [136]. Current low-*k* dielectrics, including oxides and organics, exhibit higher *k* values and possess weaker thermal and mechanical properties [97, 98, 137]. Ultra-low-k a-BN has been synthesized by several groups, demonstrating superior thermal and chemical stability. Hong et al. [7] synthesized a low-k dielectric a-BN thin film that showed significant diffusion barrier properties by testing cobalt diffusion into Si. No diffusion of Co was observed, confirming the excellent diffusion barrier functionality of a-BN layers, even at high temperatures. The breakdown field of a-BN is 7.3 MV cm^−1^, nearly twice that of hBN. The a-BN film exhibited a very low leakage current density of 6.27 µA cm^−2^, which is promising for 3-nm-node device applications. Additionally, innovative capping layers for copper interconnects were fabricated using the PECVD method [138]. These layers demonstrated significant electrical properties such as low leakage current density and high breakdown field, comparable to traditional SiN blocking layers. Moreover, with exceptional thermal stability at high temperatures, the a-BN film is suitable for both back-end-of-line (BEOL) and front-end-of-line (FEOL) processes. Furthermore, simulations show that the RC delay could be reduced by approximately 10%, demonstrating the potential of a-BN capping layers for metal interconnects in integrated circuits.

#### Protection layer

Atomically thin 2D devices are sensitive to oxygen and moisture in the environment. Consequently, stability in electronic and device performance is a crucial challenge for the advancement of 2D-material electronics [15, 139]. The materials and electronic properties of 2D channels degrade when exposed to the external environment [40]. Furthermore, silanol groups on the SiO_2_ substrate surface cause interfacing interference and localized charge accumulation [42]. Encapsulation is an excellent method to mitigate these adverse effects and enhance the stability and reliability of 2D nanoelectronics. Research has demonstrated that the mobility of GFETs on an a-BN substrate increases both hole and electron mobilities [140] and enhances device performance [2]. In this context, Lu et al. reported the direct synthesis of a-BN films on various 2D substrates, employing a-BN as a passivation and heat protection layer for 2D layers [139]. It has been shown that a transfer-free, low-temperature BN capping technique ensures a good interface between the 2D channel and a-BN thin film, thereby enhancing carrier mobility and reducing current fluctuations. Another noteworthy application of a-BN was documented by Sattari-Esfahlan and coworkers [15], showcasing the robust encapsulation properties and stabilization roles of low-k a-BN in electronic device performance (Fig. 6f)). A low-temperature grown a-BN layer was employed as a double-sided (sandwich) protective layer to preserve the electronic and material properties of high-quality graphene FET (GFET) devices under humid atmospheric conditions. Raman spectroscopy confirmed that graphene quality was maintained even after extended storage at ambient conditions. Their observations revealed an approximately 26-fold enhancement in carrier mobility after sandwiching 2D graphene between a-BN films (see Fig. 6f). Both findings significantly advance the integration of a-BN into CMOS-compatible chips.

#### Gate insulator layer

General selection criteria for suitable gate insulators in FETs include low gate leakage current, effective channel control over applied gate voltage, minimal defect density, and high dielectric stability (e.g., with a breakdown field > 10 MV cm^−1^), as well as high room-temperature mobility in the semiconductor (> 200 cm^2^V^−1^ s^−1^) [142]. Traditional 3D insulators such as SiO_2_ or HfO_2_ are known to exhibit significant impurities and dangling bonds at the interface with 2D channels, leading to interfacial defects that substantially reduce mobility. Additionally, minimizing defect density is crucial to achieving the desired subthreshold swing (SS), negligible hysteresis, and minimal long-term drifts for reliable FET operations [141, 143]. Both h-BN and a-BN have demonstrated clean interfaces with 2D materials [15, 23]. Furthermore, the defect density within the BN film can vary depending on the growth methods, which is essential for minimizing gate leakage current in FET devices.

h-BN typically achieves an equivalent oxide thickness (EOT) of less than 1.32 nm, nearing the ideal ~ 1 nm EOT required for next-generation FETs. In contrast, the minimum reported EOT for a-BN is around 4.3 nm, which remains too high for advanced gate insulator applications and requires further reduction. Minimizing EOT is crucial for improving gate control, as thicker insulators weaken electrostatic coupling and contribute to short-channel effects. A lower EOT enhances device scalability, reduces leakage currents, and improves overall performance. Further advancements in optimizing a-BN’s dielectric properties and integration methods are needed to enhance its suitability as a gate insulator in future technologies.

#### Tunneling barrier

Due to its outstanding dielectric properties, BN (h-BN and potentially a-BN) is suitable for use as a tunneling barrier when positioned between two distinct 2D layers. Tunneling can occur through localized electronic states caused by defects in the BN barrier layer [144–148]. This heterostructure enables various device applications such as 2D-based resonant tunneling diodes (RTDs) [149, 150] and random-access memories (RAMs) [151, 152] where the former utilizes a 2D/BN/2D configuration and the latter benefits from Metal/BN/Metal structures (also, see Sect. 4.1.1.). The current in RAM devices can be adjusted by controlling the defect density in the a-BN film to enhance the device's performance. For example, addressing N deficiency is a significant challenge in growing nitride films by sputtering. By promoting atomic diffusion of N from Co–N created through the deposition of a BN overlayer, Ichinose et al. [151] improved the insulating properties of a-BN by enhancing surface quality and reducing pinholes. With multi-junction (MTJ) stacking structures, a negative tunnel magnetoresistance (TMR) was observed at room temperature. Additionally, 2D/BN in-plane heterostructures have demonstrated promising electrical characteristics, such as the ability to modulate the electrical conductivity of the device based on the ratio of graphene to insulating BN [154], making them potentially useful in a broad range of device applications.

### Energy efficient devices

BN materials such as hBN exhibited a high proton conductivity [153, 154] and suitable thermal conductivity, and they are an efficient component of hybrid electrodes and separators for energy storage devices [155]. With the ascending development of 2D materials, emerging properties and potential applications in energy device applications have been recently explored for BN [156, 157]. Notably, hBN is implemented to boost the performance of batteries [158, 159], solar cells (SCs) [160], energy conversion [161], and other energy harvesting platforms [162]. Recently, a-BN layers were implemented to enhance the photovoltaic performance of Cu_2_ZnSn(S, Se)_4_ solar cell (SC) using a modification of the electrode interface [161]. The suitably thick a-BN flakes were deposited by magnetron sputtering to reduce the carrier recombination at the back electrode by tuning energy band alignment and suppressing the formation of secondary phases. Initially, the low hole barrier (higher electron barrier) formed at the BN/CZTSSe interface can be overcome if the deposited aBN layer is discontinuous or thin enough. Thus, the transport of the photogenerated holes increases, and electrons to the Mo electrode are enhanced. These two phenomena alleviate the interfacial recombination at the back electrode of SC. The experimental and simulation study demonstrated that the power conversion efficiency (PCE) of solar cells is significantly improved by the insertion of an aBN layer, which is associated with a decrease in reverse saturation current density and a boost in open-circuit voltage and fill factor.

Another study by Fang et al. [162] demonstrated the fabrication of an innovative composite solid-state electrolyte based on aBN nanosheets as a matrix integrated with lithium bis (trifluoromethanesulfonyl) imide (LiTFSI) and polymer polyvinylidene fluoride-hexafluoropropylene copolymer (PVDF-HFP), using a simple solution-casting method. A hybrid method of ball milling and pure water exfoliation is used to produce aBN nanosheets. Besides the experimental finding, the theoretical results demonstrated that the B atoms on the surface of aBN exhibited a high adsorption energy towards O atoms in TFSI, expediting lithium salt dissociation and resulting in high ionic conductivity of the electrolyte. The aBN nanosheets with excellent insulation properties, electrochemical stability, and high lithium-ion conductivity improve the electrochemical performance of LiFePO_4_/Li battery including wide electrochemical stability window (5 V), high capacity of 128.1 mAhg^−1^, and retains 94.5% of its original capacity after 500 charge–discharge cycles.

### Potential optoelectronic application

The crystalline counterpart of a-BN (e.g., h-BN) exhibits unique optical properties that have paved the way for innovative photonic and optoelectronic functionalities. Intrinsic hyperbolic characteristics in the IR spectrum and natural defects are essential for achieving single-photon and UV emission and detection functionalities [165, 166] as well as infrared nano-photonics [164, 167]. The strong interaction between light and matter, coupled with significant band-edge absorption, facilitates the achievement of deep-ultraviolet (DUV) emission photodetection using h-BN nanostructures [168]. Consequently, there is substantial interest in the growth and application of h-BN in photonic technologies [1, 10, 12, 21, 165–168]. For instance, extremely high emission intensity has been observed, which ranks among the highest brightness levels reported for quantum emitters in the visible spectrum (see Fig. 6(g)) in the crystalline form of a-BN. The high emission intensity results from the wide band gap and defect-induced emission centers in BN, such as point defects, boron and nitrogen vacancies, and impurities. The wide band gap enables the emission of high-energy photons during electron excitation and recombination, leading to strong photoluminescence (PL) and electroluminescence (EL). Additionally, defect-induced centers introduce localized states within the band gap, serving as emission and recombination sites that enhance PL and EL, particularly in the UV range. These defects are often responsible for the intense emission observed in hBN-based materials. Moreover, strong spin–orbit coupling further boosts emission intensity by enabling efficient interaction between an electron’s spin and orbital motion, leading to enhanced photon emission. Now that a single defect has been successfully introduced in h-BN [165], there is potential for creating single defects in a-BN films which may be beneficial for similar light emitter applications. Recently, a high-performance UV photodetector based on a-BN thin film, grown by magnetron sputtering at low temperatures, was demonstrated [168]. This device exhibits a low dark current and a high photo-to-dark ratio with exceptional spectral selectivity in the vacuum-ultraviolet (VUV) band. Both the high material purity and compact composition of the produced BN films show great promise for UV photodetection applications (Fig. 6(h)). Considering all these advantages, one can conclude that BN (e.g., both h-BN and a-BN) will be a pivotal material for future applications in photo-related technologies.

Table 1 summarizes the recent growth techniques of a-BN, representative electrical properties, critical material parameters, and growth details. ALD-grown a-BN has shown high potential for developing high-k films at low temperatures. However, its application in high-performance devices, such as substrates and gate-insulating layers, requires improved film morphology. Additionally, oxygen contamination at low temperatures can adversely impact film properties, increasing defect concentrations like vacancies and negatively influencing leakage current in high operational fields. On the other hand, CVD has demonstrated potential for large-scale film growth under CMOS-compatible conditions, employing low temperatures and a transfer-free growth approach.

**Table 1:** List of important a-BN growth and related parameters

Ref	Dielectric constant	Breakdown field (MV/cm)	Bandgap (eV)	Growth method	Growth temperature (˚C)	Growth substrate	a-BN role
[169]* Thin Solid Films* 571, 51 (2014)	–	–	5.65–5.83	ALD	500–900	Si, Al_2_O_3_	–
[43]* 2D Mater* 5, 011011 (2018)	Low	–	1–8.5	PLD	200	–	–
[7]* Nature* 582, 511 (2020)	1.16–1.76	7.3	-	ICP-CVD	400	Si	Interconnect
[168]* Appl. Phys. Lett.* 117, 023504 (2020)	–	–	5.9	Sputtering	500	AL_2_O_3_	UV Photodetection
[38]* Adv. Funct. Mater.* 26, 2640 (2016)	5.25–6.55	~ 10	4.5	PLD	> 200	Oxides, 2Ds, metals, polymers	–
[40]* Adv. Mater. Technol.,* 7,2,200,022 (2022)	2	–	5.9	ICP-CVD	400	Si	Ultralow-*k* dielectric
[39]* Mater. Lett.* 227, 284 (2018)	~ 3	10	–	Sputtering	25	Si	–
[48]* J. of Non-Cryst. Solids* 198, 403 (1996)	–	2.2	5	PE-CVD	200–400	Si, glass, quartz	–
[15]* ACS Appl. Mater. Interfaces* 15, 7274 (2023)	1.2–2.3	–	–	LP-CVD	250	Graphene, Si, SiO_2_	Encapsulation, substrate
[81]* Nat. Commun.* 15, 4016 (2024)	4.3–8.6	4.4–8.2	5.6–6	ALD	60–250	Si	Multi-functional device

## Outlook and perspectives

a-BN, with its distinctive advantages over crystalline BN materials, presents new opportunities for enhancement and practical implementation in next-generation electronics and optoelectronics. This review has provided a comprehensive overview of a-BN’s unique optical and electrical properties, recent advances in its synthetic methods, and its various applications.

While a-BN has demonstrated potential in high-performance electronic components, several challenges remain; first, understanding the detailed structure–property relationships, encompassing chemical, mechanical, electrical, and optical properties, is essential for its widespread application across various domains. In recent years, significant progress has been made in understanding the structural dynamics of amorphous 2D materials. However, many questions still persist regarding the role of defects and their impact on a-BN properties. The properties of a-BN can vary widely, influenced by the degree of amorphization and chemical composition, which are strongly dictated by synthesis conditions. Therefore, systematic preparation tailored to these variations is essential, along with a concurrent approach of characterization techniques and theoretical calculations.

Another major issue is associated with the integration of a-BN with other bulk and 2D materials. Previous reports have demonstrated the potential of a-BN as a functional layer in various device structures, yet technical challenges persist. When integrating a-BN with bulk materials used in standard CMOS processes, the adoption of 3D architectures in recent CMOS technologies necessitates addressing the formation of uniform a-BN layers on bulk materials with complex geometries, such as 3D structures with high aspect ratios. When integrating with 2D materials, maintaining a sharp interface between layers is crucial because it determines their device performance. The recently employed polymer-based physical stacking and transfer technique presents challenges in forming conformal contact and may leave nanoscale residue as an interfacial contaminant, potentially compromising the device's reliability. To address these challenges, developing a direct a-BN growth technique could be a potential solution, ensuring enhanced interface quality for optimal device performance. Furthermore, while ALD-grown a-BN provides high dielectric films at low temperatures, the implementation of films in high-performance device applications, such as substrate and gate-insulating layers, should enhance film morphology and reduce defect concentrations like vacancies. On the other hand, CVD shows promise for larger film growth under CMOS-compatible conditions using a low temperature and transfer-free growth approach.

## Data Availability

The review is based on the published data and sources of data upon which conclusions can be found in the reference list.
